# Distribution of Non-AT_1_, Non-AT_2_ Binding of ^125^I-Sarcosine^1^, Isoleucine^8^ Angiotensin II in Neurolysin Knockout Mouse Brains

**DOI:** 10.1371/journal.pone.0105762

**Published:** 2014-08-22

**Authors:** Robert C. Speth, Eduardo J. Carrera, Catalina Bretón, Andrea Linares, Luz Gonzalez-Reiley, Jamala D. Swindle, Kira L. Santos, Ines Schadock, Michael Bader, Vardan T. Karamyan

**Affiliations:** 1 Department of Pharmaceutical Sciences, Nova Southeastern University, Fort Lauderdale, Florida, United States of America; 2 Department of Physiology and Functional Genomics, University of Florida, Gainesville, Florida, United States of America; 3 Farquhar College of Arts and Sciences, Nova Southeastern University, Fort Lauderdale, Florida, United States of America; 4 College of Dentistry, University of Florida, Gainesville, Florida, United States of America; 5 Max-Delbrück-Center for Molecular Medicine, Berlin, Germany; 6 Department of Pharmaceutical Sciences, Texas Tech University Health Sciences Center, Amarillo, Texas, United States of America; 7 Center for Blood-Brain Barrier Research, Texas Tech University Health Sciences Center, Amarillo, Texas, United States of America; University of Wurzburg, Germany

## Abstract

The recent identification of a novel binding site for angiotensin (Ang) II as the peptidase neurolysin (E.C. 3.4.24.16) has implications for the renin-angiotensin system (RAS). This report describes the distribution of specific binding of ^125^I-Sarcosine^1^, Isoleucine^8^ Ang II (^125^I-SI Ang II) in neurolysin knockout mouse brains compared to wild-type mouse brains using quantitative receptor autoradiography. In the presence of p-chloromercuribenzoic acid (PCMB), which unmasks the novel binding site, widespread distribution of specific (3 µM Ang II displaceable) ^125^I-SI Ang II binding in 32 mouse brain regions was observed. Highest levels of binding >700 fmol/g initial wet weight were seen in hypothalamic, thalamic and septal regions, while the lowest level of binding <300 fmol/g initial wet weight was in the mediolateral medulla. ^125^I-SI Ang II binding was substantially higher by an average of 85% in wild-type mouse brains compared to neurolysin knockout brains, suggesting the presence of an additional non-AT_1_, non-AT_2_, non-neurolysin Ang II binding site in the mouse brain. Binding of ^125^I-SI Ang II to neurolysin in the presence of PCMB was highest in hypothalamic and ventral cortical brain regions, but broadly distributed across all regions surveyed. Non-AT_1_, non-AT_2_, non-neurolysin binding was also highest in the hypothalamus but had a different distribution than neurolysin. There was a significant reduction in AT_2_ receptor binding in the neurolysin knockout brain and a trend towards decreased AT_1_ receptor binding. In the neurolysin knockout brains, the size of the lateral ventricles was increased by 56% and the size of the mid forebrain (−2.72 to +1.48 relative to Bregma) was increased by 12%. These results confirm the identity of neurolysin as a novel Ang II binding site, suggesting that neurolysin may play a significant role in opposing the pathophysiological actions of the brain RAS and influencing brain morphology.

## Introduction

The classical renin-angiotensin system (RAS) was initially characterized as a major regulator of systemic blood pressure and fluid and electrolyte balance by way of direct vasoconstriction of vascular smooth muscle, generalized sympathetic nervous system activation, and mediation of aldosterone and epinephrine release [1]–[6]. The RAS is presently known to be comprised of circulating angiotensins and independent tissue-specific RASs [7]–[9]. Prominent among tissue-specific RASs is the brain RAS [10]–[12]. Angiotensin (Ang) II, the main effector peptide of the RAS, is abundantly expressed in the brain [13], [14]. There are two primary G protein-coupled receptors for Ang II reported to be present in the brain: type 1 (AT_1_) and type 2 (AT_2_) [15]–[17]. The AT_1_ receptor mediates the classical functions noted above [18] along with thirst and sodium chloride appetite [19], [20]. This receptor may also be associated with diabetes, depression, Parkinson's disease, and Alzheimer's disease [12]. The AT_2_ receptor is believed to act antagonistically to the AT_1_ receptor by mediating vasodilation and cerebroprotection, as well as neural differentiation, regeneration, and neurotrophic actions [21]–[24].

**Figure 1:**
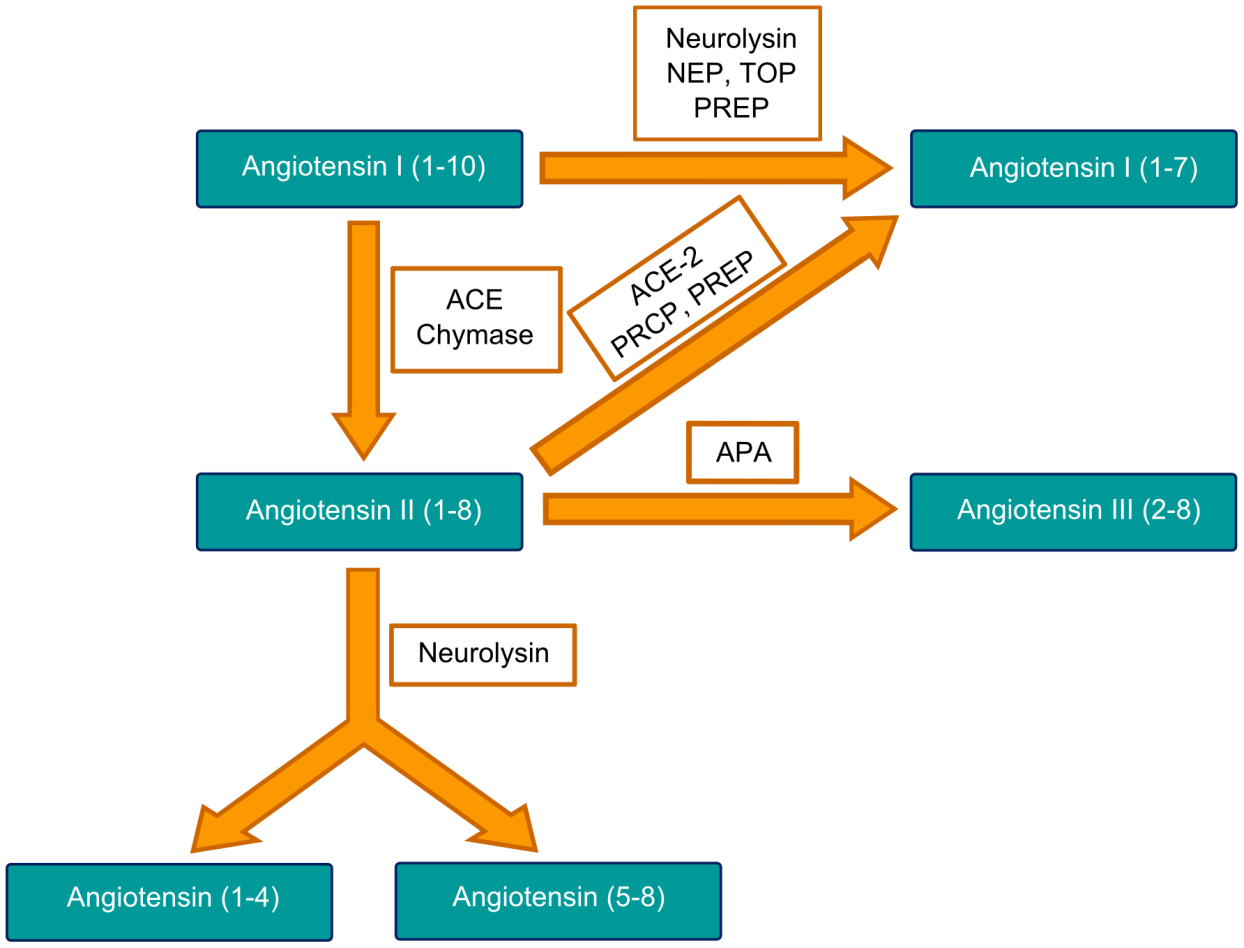
Metabolic pathways of Ang peptides. Metabolic routes of Ang I and II by neurolysin and other peptidases of the RAS. ACE =  angiotensin-converting enzyme, dipeptidyl carboxypeptidase I, Kininase II, EC 3.4.15.1, CD143; ACE-2 =  angiotensin-converting enzyme-2, EC 3.4.17.23; APA =  aminopeptidase A, glutamyl aminopeptidase, EC 3.4.11.7, CD249; NEP =  neprilysin, neutral endopeptidase, EC 3.4.24.11; PRCP =  prolyl carboxypeptidase, angiotensinase C, carboxypeptidase P, EC 3.4.16.2; PREP =  prolyl endopeptidase, post-prolyl cleaving enzyme, EC 3.4.21.26; TOP =  thimet oligopeptidase, EC 3.4.24.15. Adapted from Wright et al. [12].

There are several biochemical pathways for the breakdown of Ang II into inactive peptides (Figure 1). Ang II can be converted to the short-lived heptapeptide Ang III by glutamyl aminopeptidase-A. Ang III is then cleaved by the membrane-bound alanyl aminopeptidase-N to form the 3–8 hexapeptide Ang IV [25]. Further metabolism of Ang IV by aminopeptidases results in inactive peptides [26], [27]. Ang II can also be metabolized by a variety of mono- and di-peptidyl aminopeptidases [27]. Alternatively, Ang II can be converted to Ang (1–7) by angiotensin-converting enzyme-2 (ACE-2), prolyl carboxypeptidase [28] and prolyl endopeptidase [29], [30], see reviews [12], [27]. Ang (1–7) has been of particular interest lately as its actions through the G protein-coupled receptor Mas serve to counterbalance the deleterious effects of Ang II [31], [32]. Actions of Ang (1–7) are associated with vasodilation and cardioprotection, as well as decreased hypertrophy, fibrosis, and thrombosis [32]. Further aminopeptidase activity on Ang (1–7) produces Ang (2–7) and Ang (3–7), which may also have biological activity [33]–[35].

A new dimension was added to the brain RAS with the discovery of a novel non-AT_1_, non-AT_2_ binding site for Ang II [36]. Initial studies of this novel binding site could not ascertain its function and it was hypothesized to be either a signaling or clearance receptor, or a peptidase [37]–[39]. We recently reported the metalloendopeptidase neurolysin (EC 3.4.24.16, also known as microsomal endopeptidase or mitochondrial oligopeptidase) to be the novel non-AT_1_, non-AT_2_ Ang II binding site [40]. This binding site is unmasked by p-chloromercuribenzoic acid (PCMB) which is an organomercurial compound that inhibits the activity of numerous enzymes, including neurolysin [41]. Most likely, PCMB causes a conformational change in neurolysin that enhances its ability to bind angiotensins, but inhibits its ability to cleave these substrates. The density of this binding site in the brain is substantially higher than that of AT_1_ or AT_2_ receptors in the rat brain [36], [42], [43]. While neurolysin is mostly known for its actions on neurotensin, its primary substrate, it can also metabolize Ang I to form Ang (1–7) [44], [45] and Ang II to form the inactive peptides Ang (1–4) and Ang (5–8) [45], [46].

A critical component of the study that identified neurolysin as the non-AT_1_, non-AT_2._

Ang II binding site was the use of a mouse strain in which the neurolysin gene was knocked out [40]. Expression of the non-AT_1_, non-AT_2_ binding site was dramatically decreased in the brains of the neurolysin knockout mouse strain compared to wild-type mice. Distribution was then examined using quantitative densitometric autoradiography. A qualitative sampling of this autoradiographic analysis was included in our previous publication [40]. Additionally, we examined the distribution and concentration of AT_1_ and AT_2_ receptors in the brains of the neurolysin knockout mouse strain in comparison to wild-type mice using the same methodology.

## Materials and Methods

### Ethics Statement

This study was carried out in accordance with the recommendations in the Guide for the Care and Use of Laboratory Animals of the National Institutes of Health. The animal protocols were approved by the Institutional Animal Care and Use Committee of Nova Southeastern University (IACUC Control# 014-389-09- 0922) and by the Committee on the Ethics of Animal Experiments of the State of Berlin (LAGESO, Permit Number: T0042/06).

### Animals

Six male mouse brains, 3 wild-type (WT) and 3 neurolysin knockout (KO), were collected from 12-week old adult male mice maintained in 12-hour light/dark cycle and fed ad libitum in the laboratory of Dr. Michael Bader. The neurolysin knockout mice were generated using gene-trap technology and expressed on a C57Bl/6 background [47]. Mice were sacrificed with an overdose of ketamine-xylazine anesthesia. The brains were stored at −80 °C and shipped to Nova Southeastern University on dry ice. A full characterization of the neurolysin knockout mice documenting complete loss of neurolysin protein and mRNA is described in a manuscript to be submitted for publication.

### Materials

Ang II and Sar^1^, Ile^8^ Ang II (SI-Ang II) were acquired from Phoenix Pharmaceuticals and Bachem and were radioiodinated by a previously described method [48]. Losartan was obtained from Dr. Ron Smith of Dupont Merck, PD123319 from Tocris, and PCMB sodium salt from MP Bio-medicals.

### Receptor autoradiography

Receptor autoradiographic studies were performed following established protocols [49]–[51]. Frozen mouse brains were sectioned in the coronal plane at a thickness of 20 µm, mounted on charged slides in repeating series of 6 (Table 1) air dried, and stored at −70°C. After 2 weeks (for non-AT_1_, non AT_2_ binding) or 4 months (for AT_1_ and AT_2_ binding), sections were thawed and pre-incubated in assay buffer for 30 min at room temperature. The assay buffer contained 150 mM NaCl, 5 mM EDTA, 0.1 mM bacitracin, and 50 mM NaPO_4_ at pH 7.1–7.2. For non-AT_1_, non-AT_2_ binding, this buffer also contained 150 µM PCMB. Following pre-incubation, the slide-mounted sections were incubated in the same buffer with 250 pM ^125^I-labeled Sar^1^, Ile^8^ Ang II (^125^I-SI Ang II). For non-AT_1_, non-AT_2_ binding, the assay buffer also contained 10 µM losartan, 10 µM PD123319 and 150 µM PCMB. Slides with adjacent sections were incubated with 250 pM ^125^I-SI Ang II in the presence of 3 µM Ang II to determine nonspecific binding. For AT_1_ and AT_2_ receptor binding, 1 set of slides was incubated with 3 µM Ang II, an adjacent set was incubated with 10 µM PD123319, and another adjacent set was incubated with 10 µM losartan (see Table 1). After 1-hour incubation, the slides were quickly dipped in distilled water, rinsed in 5 changes of assay buffer for 15 sec each, dipped in distilled water again, and dried under a stream of cool air. Slides were mounted onto cardboard along with a ^125^I calibration standard (ARI-0133, American Radiolabeled Chemicals) and placed in an X-ray cassette. Apposed to X-ray film (Kodak MR-1) for a 38-hour exposure (for neurolysin binding) or 5-day exposure (for AT_1_ and AT_2_ receptor binding), after which the film was developed in an automated film processor.

**Table 1:** Summary of autoradiography protocol.

Grouping	Non-AT_1_, non-AT_2_		Non-specific AT_1_ and AT_2_	Total AT_1_	Total AT_2_	Histology (thionin)
	Non-specific	Total				
**Slide Series**	−1	−2	−3	−4	−5	−6
3 µM Ang II	+	-	+	-	-	-
150 µM PCMB	+	+	-	-	-	-
10 µM PD123319	+	+	-	+	-	-
10 µM losartan	+	+	-	-	+	-

Autoradiography experiments utilized PCMB, PD123319, or losartan, to unmask non-AT_1_, non-AT_2_ binding, or block AT_2_ and AT_1_ receptors, respectively. Non-radioiodinated Ang II was utilized to define specific binding of ^125^I-SI Ang II to Ang II binding sites and receptors, as described in Materials and Methods.

The sixth slide in each set of sections was Nissl-stained with thionin to histologically identify anatomical loci corresponding to brain regions in which ^125^I-SI Ang II binding was assessed (Table 1).

### Image analysis

Film images of ^125^I-SI Ang II binding to mouse brain sections were analyzed using a densitometric procedure. Films were scanned at 2400 dpi resolution. Scanned images were evaluated using an image analysis software program (MCID, Interfocus Imaging Ltd.) which quantified the ^125^I-SI Ang II binding based upon calibration with a set of ^125^I standards. A tissue equivalency of 45% was used for the calibration based upon empirical determinations (Speth, unpublished). For enhanced visualization, the black and white film images were converted to pseudocolor. To assess binding in specific brain regions, the mouse brain atlas of Franklin and Paxinos [52] was used in conjunction with visual assessment of thionin-stained brain sections and pseudocolored autoradiograms. Areas corresponding to specific brain regions were circumscribed manually and sampled densitometrically [50]. Average density and surface area values of sampled regions were recorded. To assess the expression of non-AT_1_, non-AT_2_ binding in the brains of the wild-type and neurolysin knockout strains, 32 brain regions were identified and quantitated. To assess the expression of AT_1_ and AT_2_ receptor binding, 9 and 10 brain regions were sampled, respectively.

To determine specific binding, ^125^I-SI Ang II binding not displaceable in the presence of 3 µM Ang II (nonspecific binding) was subtracted from binding in the absence Ang II (total binding) as described in Table 1. A correction was applied to normalize densitometric measurements for sections with higher background absorbance to account for variations in film background. The increased background absorbance was subtracted from density measurements in the affected sections.

The size of the lateral, third and fourth ventricles as well as the cerebral aqueduct was determined for each brain via analysis of the thionin-stained brain sections. The ventricles and aqueduct were circumscribed and the surface area for each compartment was determined at 120 micron intervals in the coronal plane. For lateral ventricles measurements of surface area were taken from ∼0.9 mm caudal to ∼1.15 mm rostral to Bregma. For the third ventricle the surface area was measured from ∼0.9 mm to ∼0.2 mm caudal to Bregma. For the fourth ventricle measurements were taken from ∼6.66 mm to ∼5.34 mm caudal to Bregma. For the cerebral aqueduct measurements were taken from ∼4.84 mm to ∼4.24 mm caudal to Bregma.

### Statistical analysis

Sampling of brain regions involved multiple determinations at different coronal levels. The average density for total and nonspecific binding from all coronal levels sampled was determined, and specific binding was derived as described above. The areas circumscribed for each region varied to some extent based on the perceived density of ^125^I-SI Ang II binding. To assess the possible impact of size measurement differences, the area sampled was also determined for each brain region of each mouse brain. Statistical comparisons of knockout versus wild-type brains for specific binding density were made with a two-way analysis of variance (strain and region). Comparison of brain surface area was also made using a two-way analysis of variance (strain and anterior-posterior coordinate). An unpaired Student's t-test was used for comparison between neurolysin knockout and wild-type mouse brain ventricle sizes, brain surface area (in areas where ventricles were measured), and the ratio of ventricle to total brain surface area. Additionally, an a priori one-tailed, unpaired Student's t-test was run to compare non-AT_1_, non-AT_2_ binding in knockout versus the wild-type brain regions. The statistical significance level was p<0.05. Values shown are mean ± SEM.

## Results

**Figure 2:**
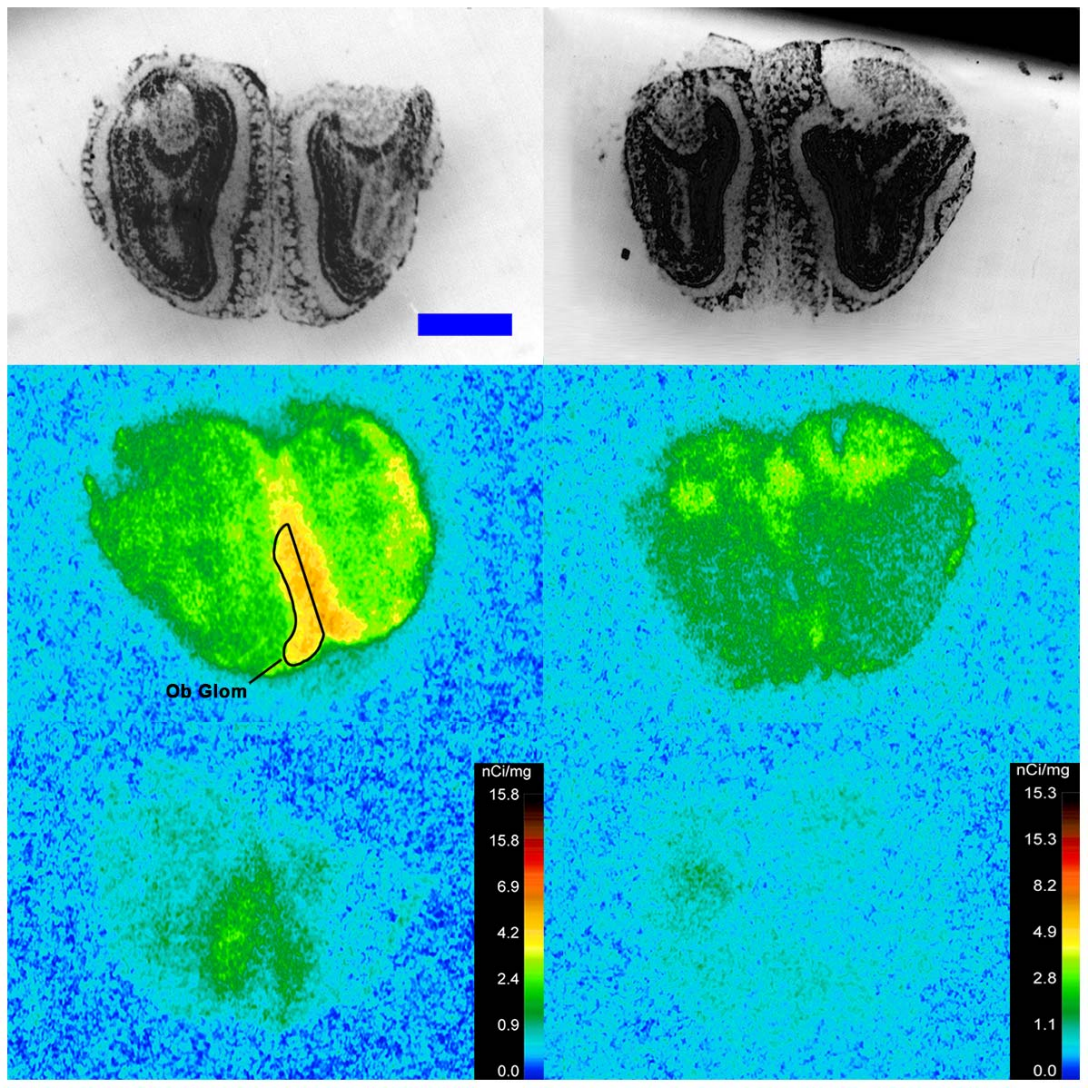
^125^I-SI Ang II binding comparison. Comparison of ^125^I-SI Ang II binding in the brains of a representative neurolysin KO (right panels) and WT (left panels) mouse strain in the presence of PCMB, losartan, and PD123319. Approximate coordinates relative to Bregma: +3.56 mm for WT and +3.32 mm for KO. Top row shows thionin-stained coronal sections adjacent to the sections used to generate the autoradiograms for “total” (middle panels) and “non-specific” (lower panels) of ^125^I-SI Ang II binding. Binding is represented in pseudocolor. The vertical calibration bar represents the relationship between ^125^I-SI Ang II binding density and the color spectrum. The blue horizontal calibration bar shown in the upper left panel  = 1 mm. This pattern is repeated for Figures 3–12.

**Figure 3:**
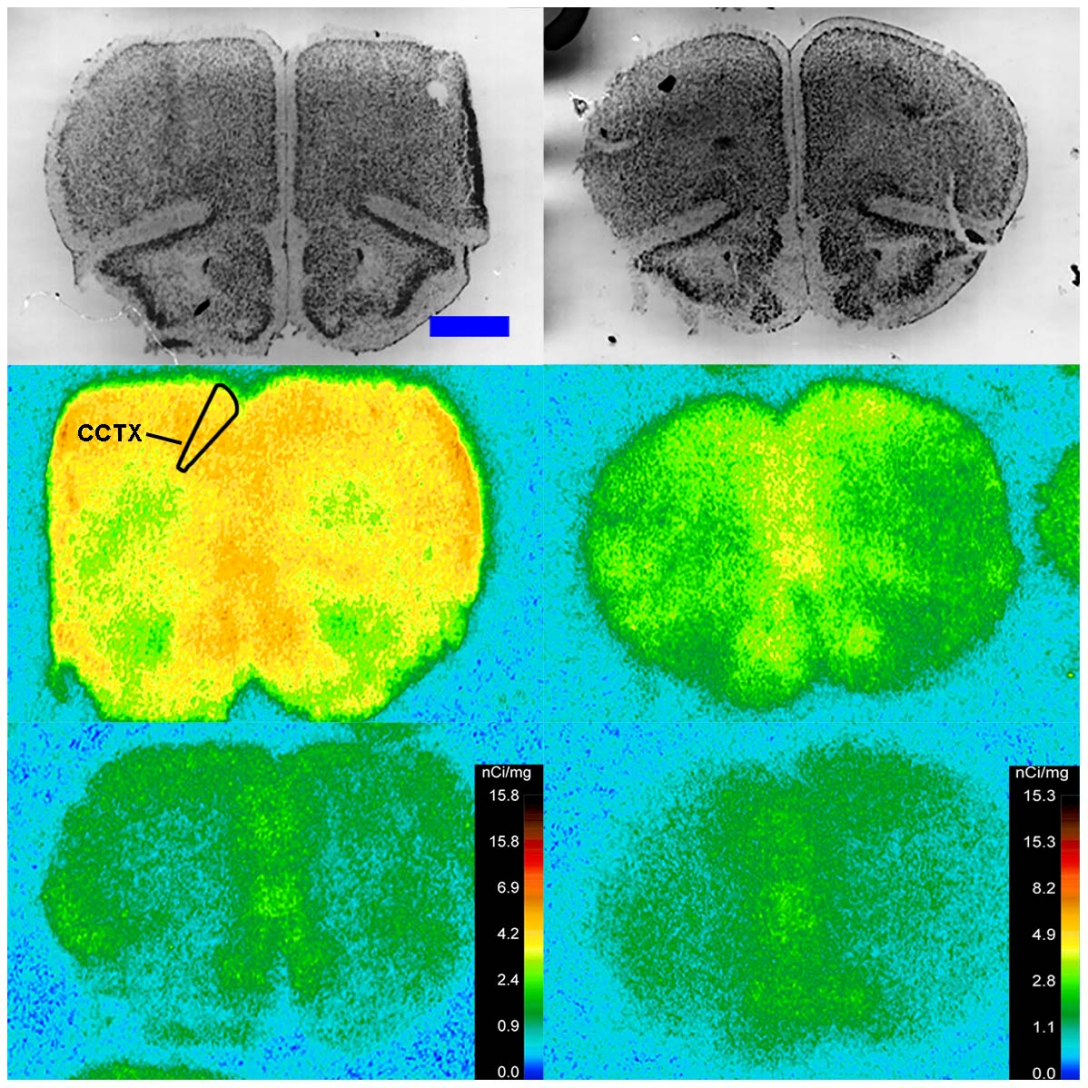
^125^I-SI Ang II binding comparison. Comparison of ^125^I-SI Ang II binding in the brains of neurolysin KO and WT mouse strains in the presence of PCMB, losartan, and PD123319. Bregma +2.22 mm (histology) and +2.10 (autoradiograms) for KO, and Bregma +2.16 (histology), +1.92 (total) and +2.04 mm (non-specific) sections for WT.

**Figure 4:**
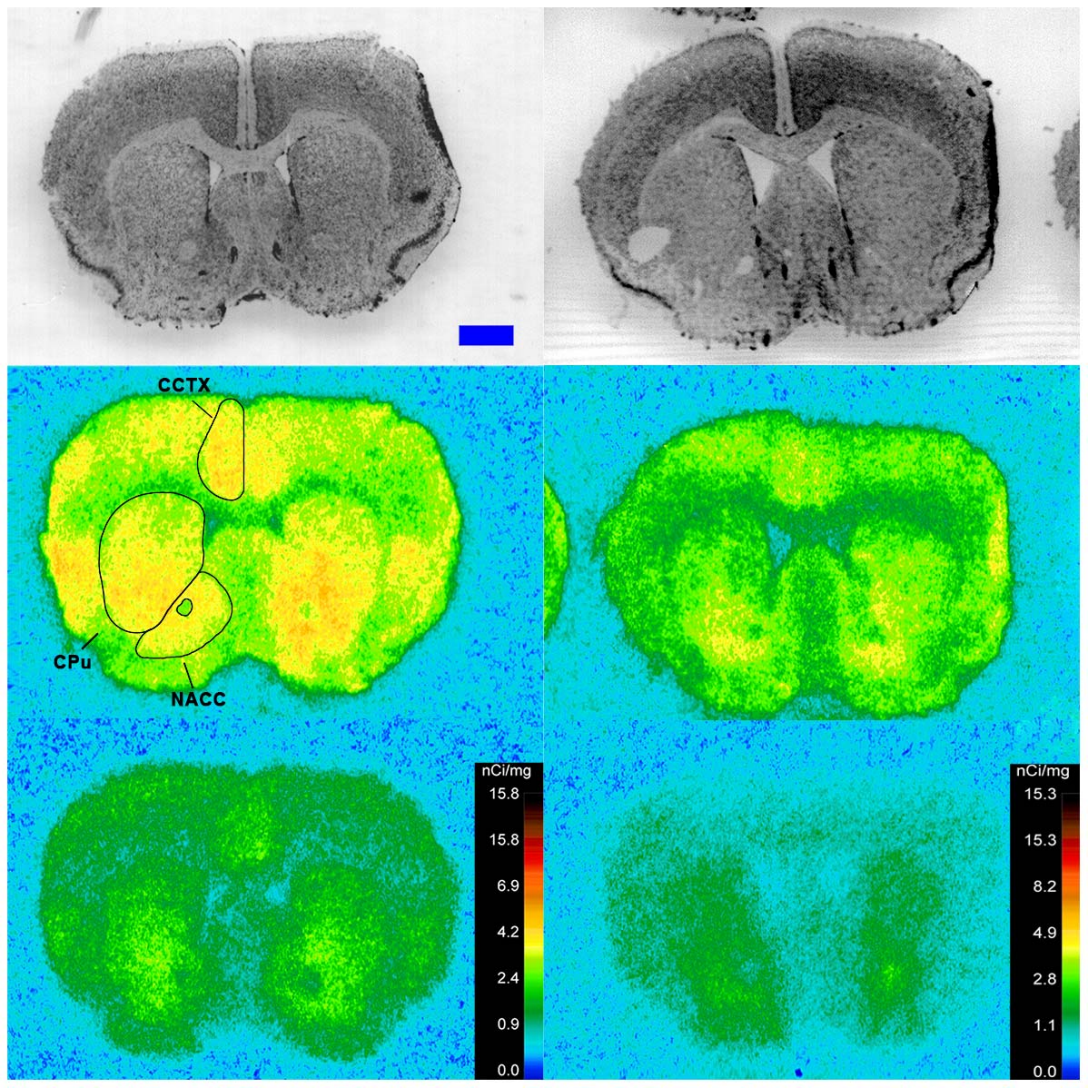
^125^I-SI Ang II binding comparison. Comparison of ^125^I-SI Ang II binding in the brains of neurolysin KO and WT mouse strains in the presence of PCMB, losartan, and PD123319. Bregma +0.86 (histology) and +0.98 mm (autoradiograms) for KO, and Bregma +0.96 mm (histology and autoradiogram) sections for WT.

**Figure 5:**
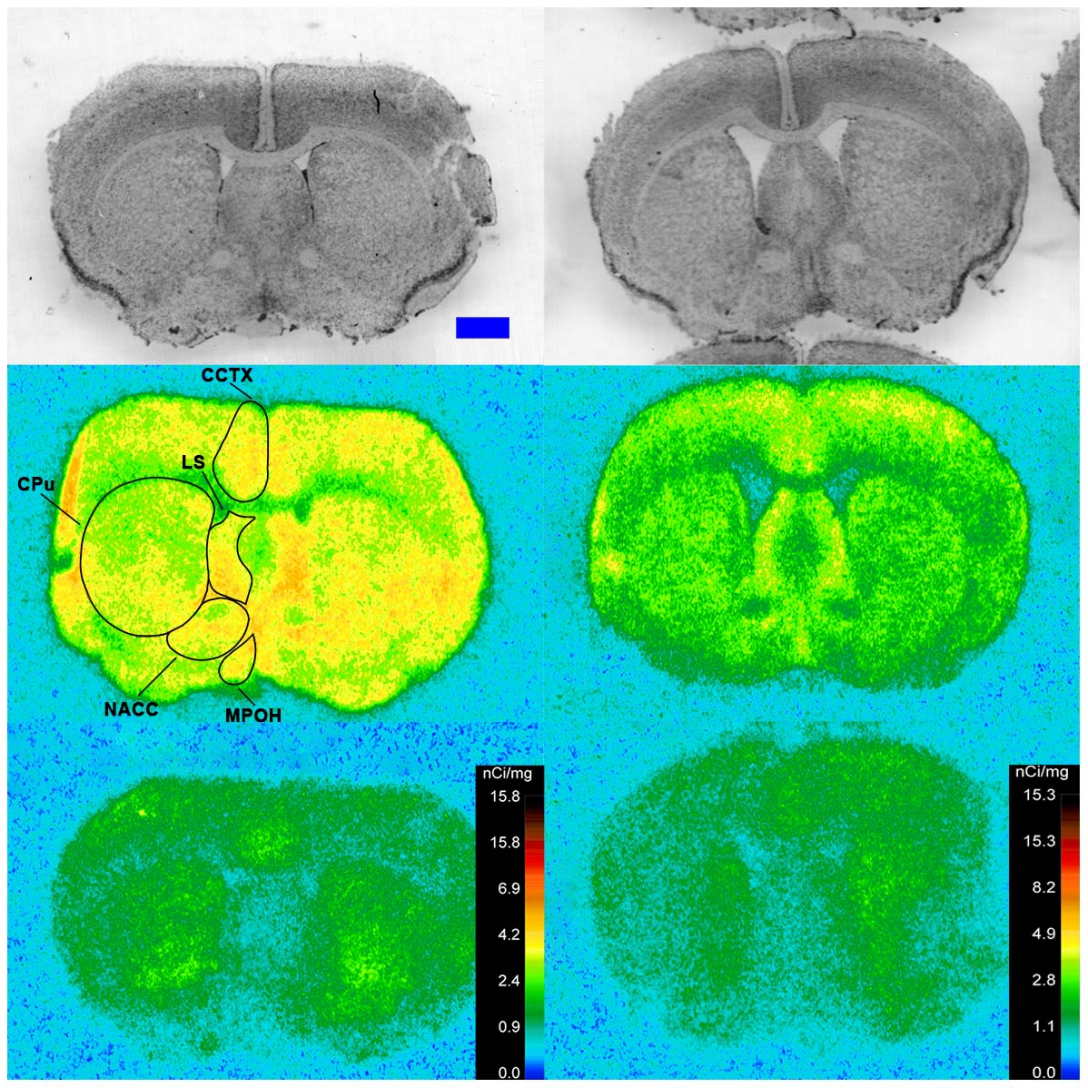
^125^I-SI Ang II binding comparison. Comparison of ^125^I-SI Ang II binding in the brains of neurolysin KO and WT mouse strains in the presence of PCMB, losartan, and PD123319. Bregma +0.38 (histology) and +0.5 mm (autoradiograms) for KO, and Bregma +0.36 mm (histology and autoradiogram) sections for WT.

**Figure 6:**
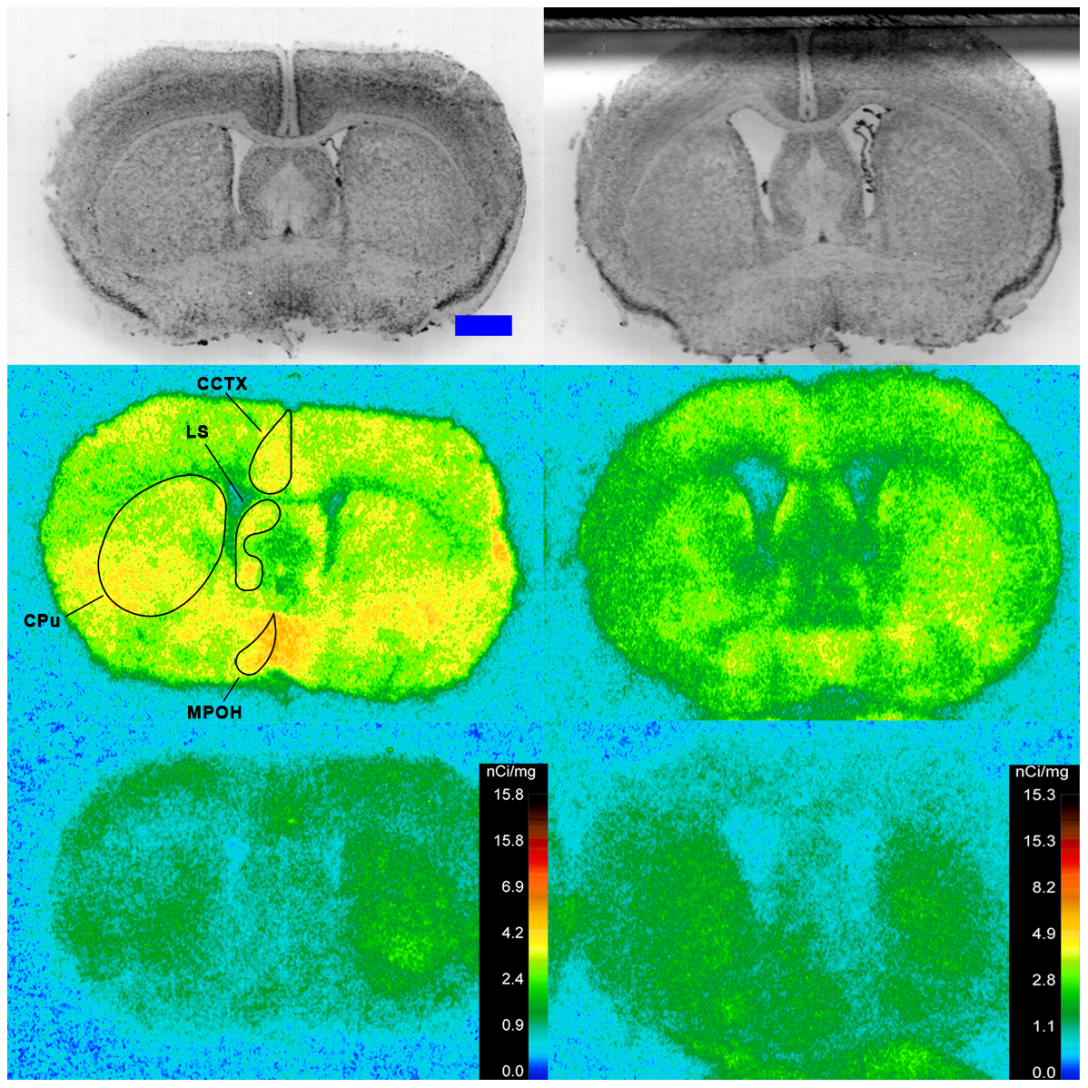
^125^I-SI Ang II binding comparison. Comparison of ^125^I-SI Ang II binding in the brains of neurolysin KO and WT mouse strains in the presence of PCMB, losartan, and PD123319. Bregma +0.08 mm for the KO and WT histological and autoradiogram sections.

**Figure 7:**
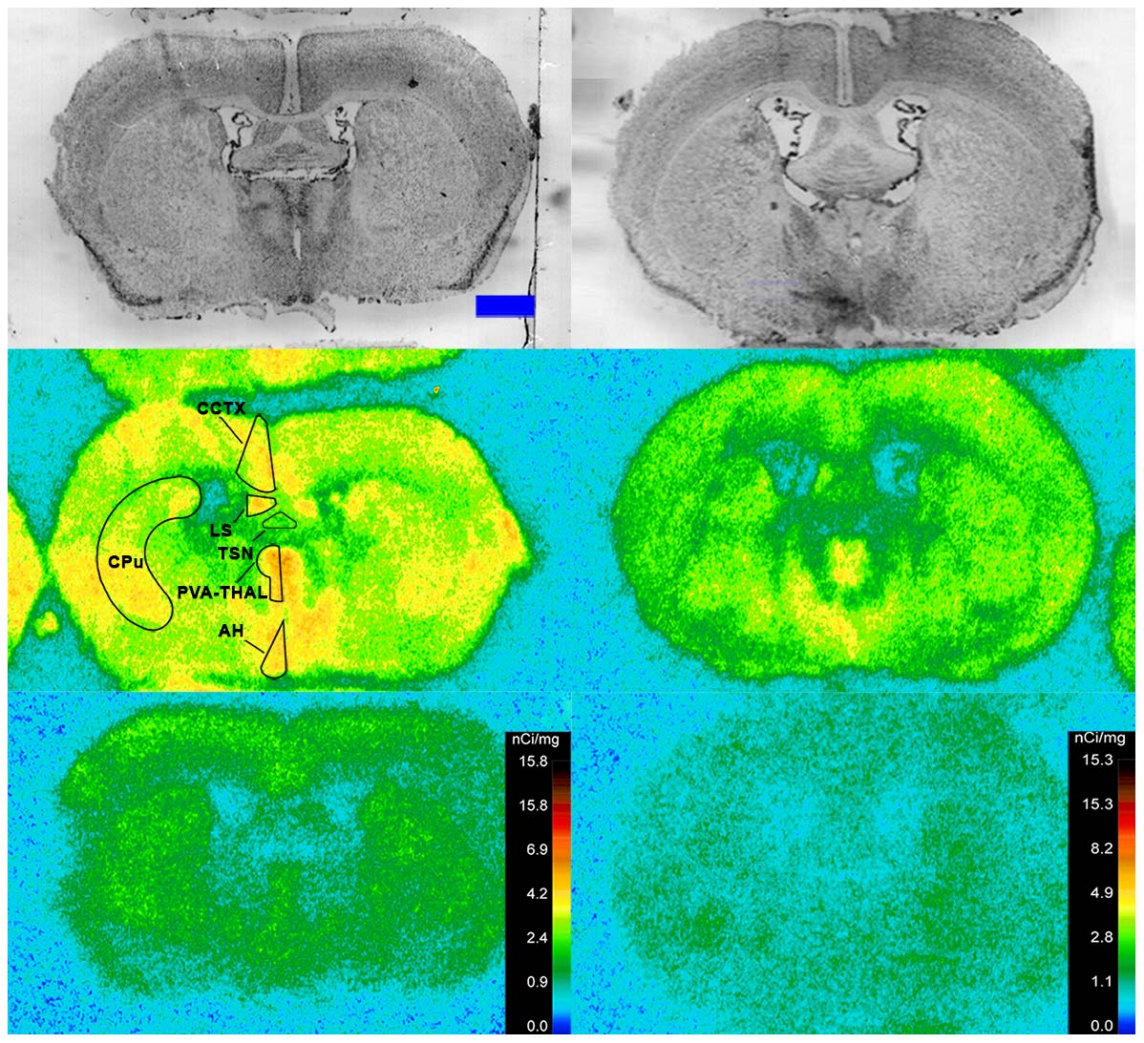
^125^I-SI Ang II binding comparison. Comparison of ^125^I-SI Ang II binding in the brains of neurolysin KO and WT mouse strains in the presence of PCMB, losartan, and PD123319. Bregma −0.34 mm for the KO and WT histological and autoradiogram sections.

**Figure 8:**
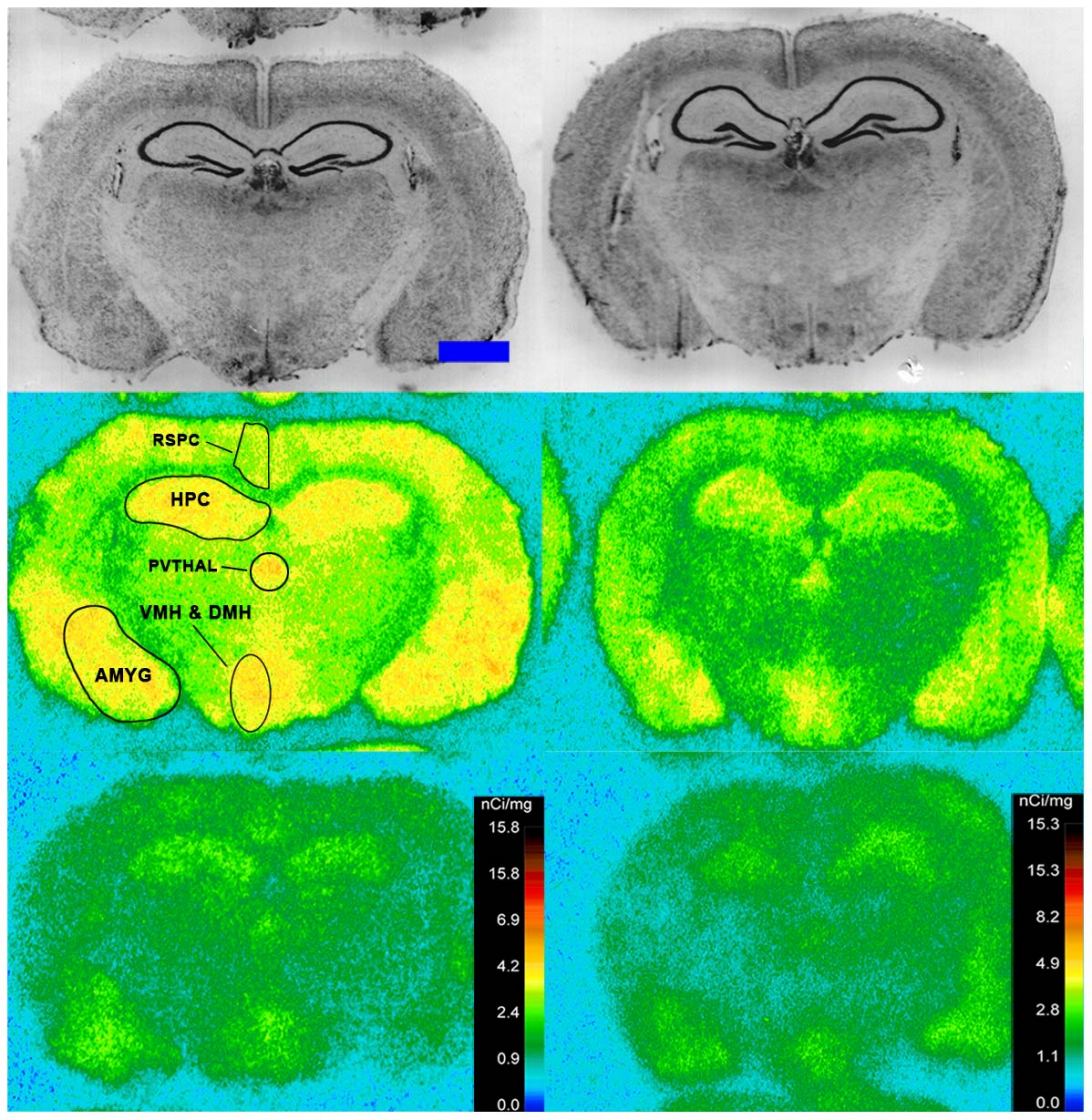
^125^I-SI Ang II binding comparison. Comparison of ^125^I-SI Ang II binding in the brains of neurolysin KO and WT mouse strains in the presence of PCMB, losartan, and PD123319. Bregma −1.82 mm for KO, and Bregma −1.70 mm for WT histological and autoradiogram sections.

**Figure 9:**
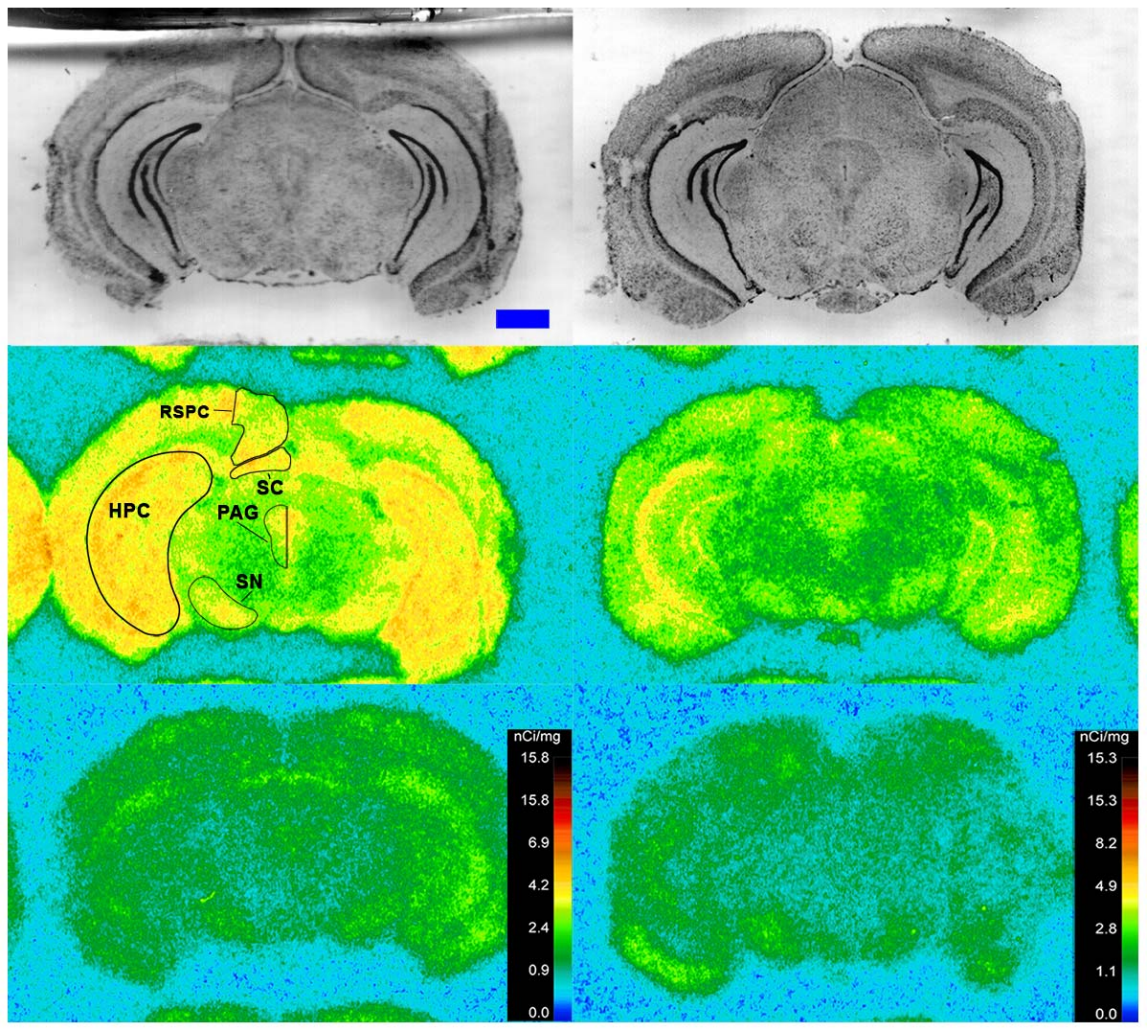
^125^I-SI Ang II binding comparison. Comparison of ^125^I-SI Ang II binding in the brains of neurolysin KO and WT mouse strains in the presence of PCMB, losartan, and PD123319. Bregma −3.40 mm (histology and autoradiograms) for KO, and Bregma −3.32 (histology) and −3.20 mm (autoradiogram) sections for WT.

**Figure 10:**
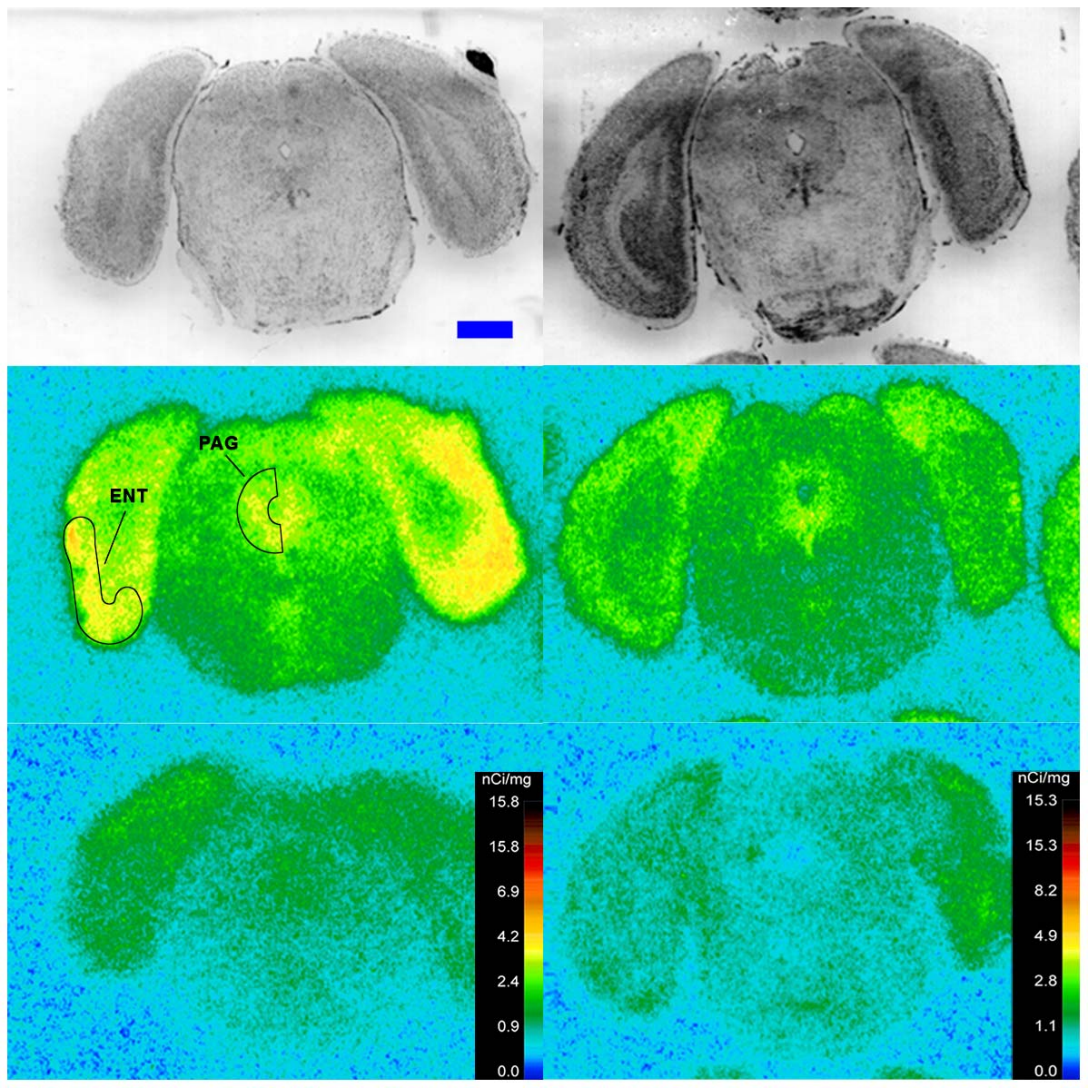
^125^I-SI Ang II binding comparison. Comparison of ^125^I-SI Ang II binding in the brains of neurolysin KO and WT mouse strains in the presence of PCMB, losartan, and PD123319. Bregma −4.24 mm (histology and autoradiograms) for KO, and Bregma −4.36 (histology) and −4.24 mm (autoradiogram) sections for WT.

**Figure 11:**
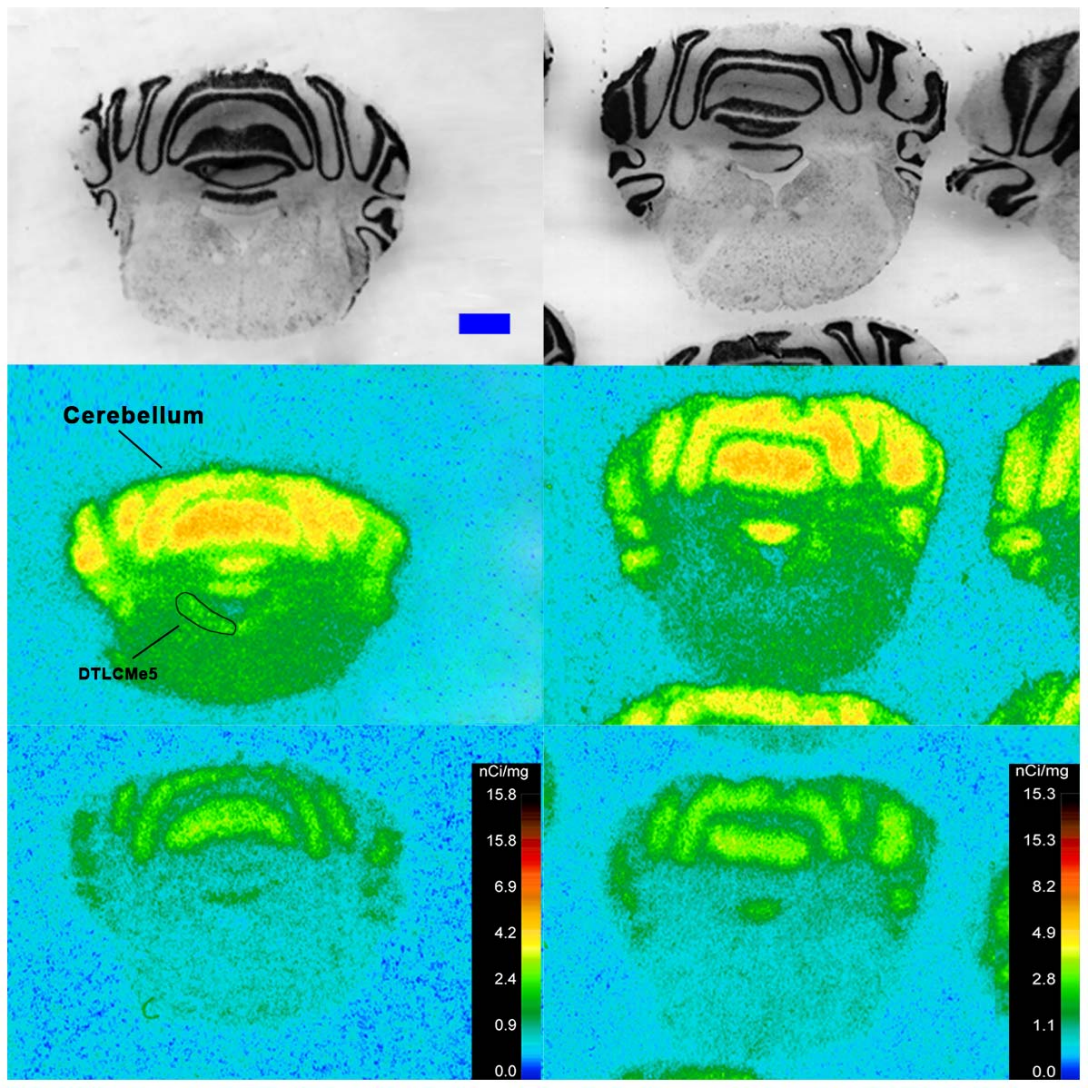
^125^I-SI Ang II binding comparison. Comparison of ^125^I-SI Ang II binding in the brains of neurolysin KO and WT mouse strains in the presence of PCMB, losartan, and PD123319. Bregma −5.8 mm for the KO and WT histological and autoradiogram sections.

**Figure 12:**
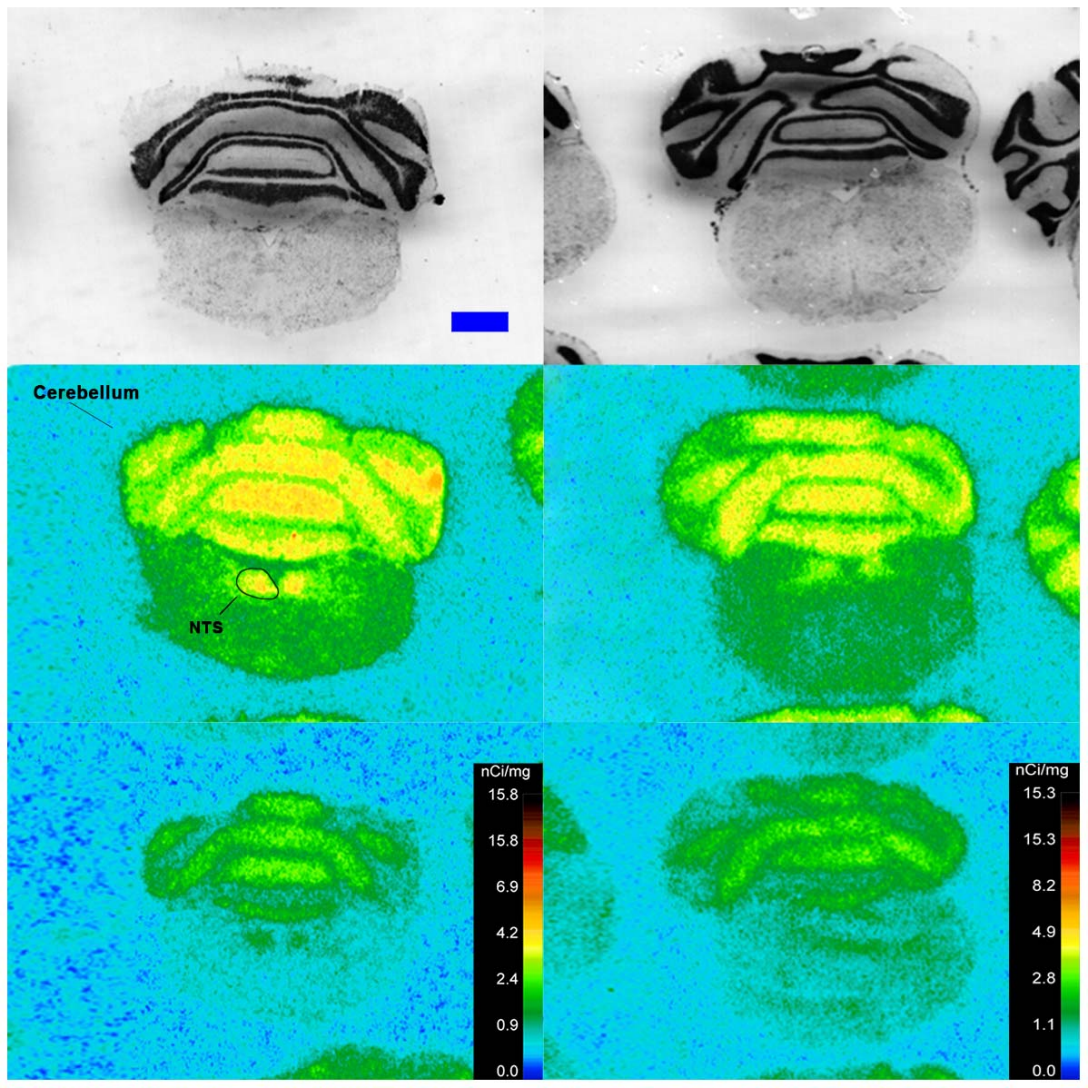
^125^I-SI Ang II binding comparison. Comparison of ^125^I-SI Ang II binding in the brains of neurolysin KO and WT mouse strains in the presence of PCMB, losartan, and PD123319. Bregma −7.2 mm for the KO and WT histological and autoradiogram sections.

**Figure 13:**
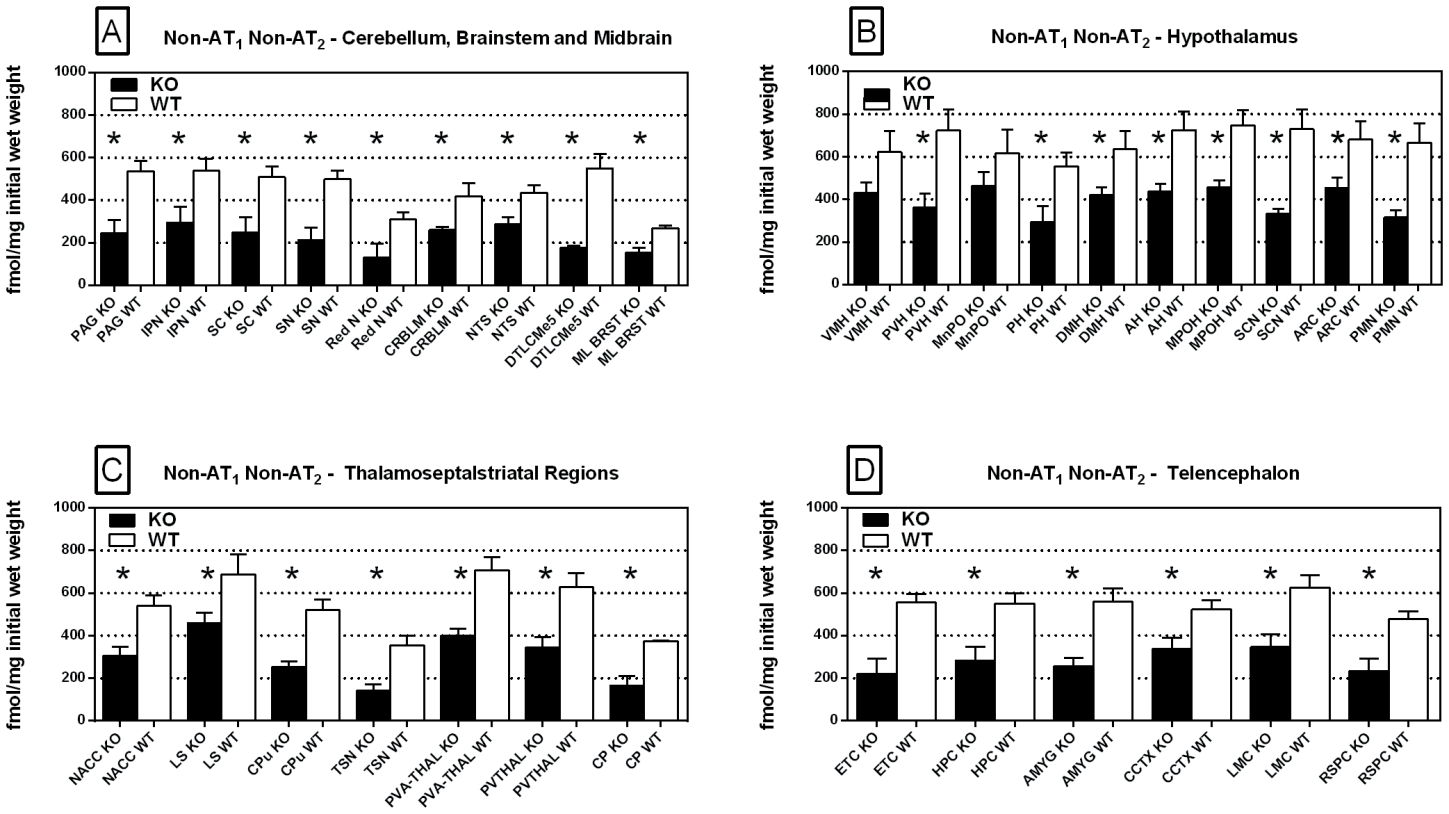
Regional distribution: non-AT_1_, non-AT_2_ binding. Regional distribution of non-AT_1_, non-AT_2_ Ang II binding in neurolysin KO and WT mouse brains. Brain regions were divided into cerebellum, brainstem and midbrain (Panel A), hypothalamic nuclei (Panel B), thalamoseptalstriatal regions (Panel C), and telencephalic regions (Panel D). In all but two regions, a priori t-tests showed significant reduction in ^125^I-SI Ang II binding in the brains of the neurolysin KO mice. * p<0.05. AH, Anterior Hypothalamus; AMYG, Amygdala; ARC, Arcuate Nucleus; CCTX, Cingulate Cortex; CP, Choroid Plexus; CPu, Caudate Putamen; CRBLM, Cerebellum; DMH, Dorsomedial Hypothalamus; DTLCMe5, Dorsal Tegmentum, Locus Coeruleus and Mesencephalic Nucleus of the Trigeminal Nerve; ETC, Entorhinal Cortex; HPC, Hippocampus; IPN, Interpeduncular Nucleus; LMC, Limbic Cortex; LS, Lateral Septum; ML BRST, Mediolateral Brain Stem; MnPO, Median Preoptic Nucleus; MPOH, Medial Preoptic Nucleus; NACC, Nucleus Accumbens; NTS, Nucleus Tractus Solitarius; PAG, Periaqueductal Gray; PH, Posterior Hypothalamic Area; PMN, Premamillary Nucleus; PVA-THAL, Paraventricular Thalamic Nucleus, Anterior; PVH, Paraventricular Hypothalamic Nucleus; PVTHAL, Paraventricular Thalamic Nucleus; Red N, Red Nucleus; RSPC, Retrosplenial Cortex; SC, Superior Colliculus; SCN, Suprachiasmatic Nucleus; SN, Substantia Nigra; TSN, Triangular Septal Nucleus; VMH, Ventromedial Hypothalamic Nucleus.

Specific binding of ^125^I-SI Ang II in the presence of PCMB, losartan and PD123319 was observed throughout the brains of both the wild-type and neurolysin knockout mouse strains (Figures 2–12), and was measured in 32 regions (Figure 13). Two-way analysis of variance indicated a highly significant (p<0.0001) reduction of 46% in ^125^I-SI Ang II binding in the brains of the neurolysin knockout strain. There was also a highly significant (p<0.0001) regional variation in ^125^I-SI Ang II binding in both strains, as can be visually appreciated in Figures 2–12. There was no strain by region interaction (p = 0.883), indicating that the extent of the reduction in ^125^I-SI Ang II binding in the neurolysin knockout mouse strain did not vary significantly between brain regions. A priori t-tests comparing binding of ^125^I -SI Ang II in the presence of PCMB, losartan and PD123319 between strains indicated a significant (p<0.05) difference in all regions except for the ventral medial hypothalamus (VMH) and median preoptic nucleus (MnPO), as shown in Figure 13, Panel B. As can be seen in Figures 11 and 12, ^125^I-SI Ang II binding in the cerebellum was almost exclusively localized to the molecular layer; however, it was unfeasible to single out this layer in our measures. Therefore, the total surface area of the cerebellum (granular and molecular layers) was assayed. This, along with high non-specific ^125^I-SI Ang II binding in the cerebellum contributed to the relatively low specific ^125^I-SI Ang II binding reported in Figure 13.

**Figure 14:**
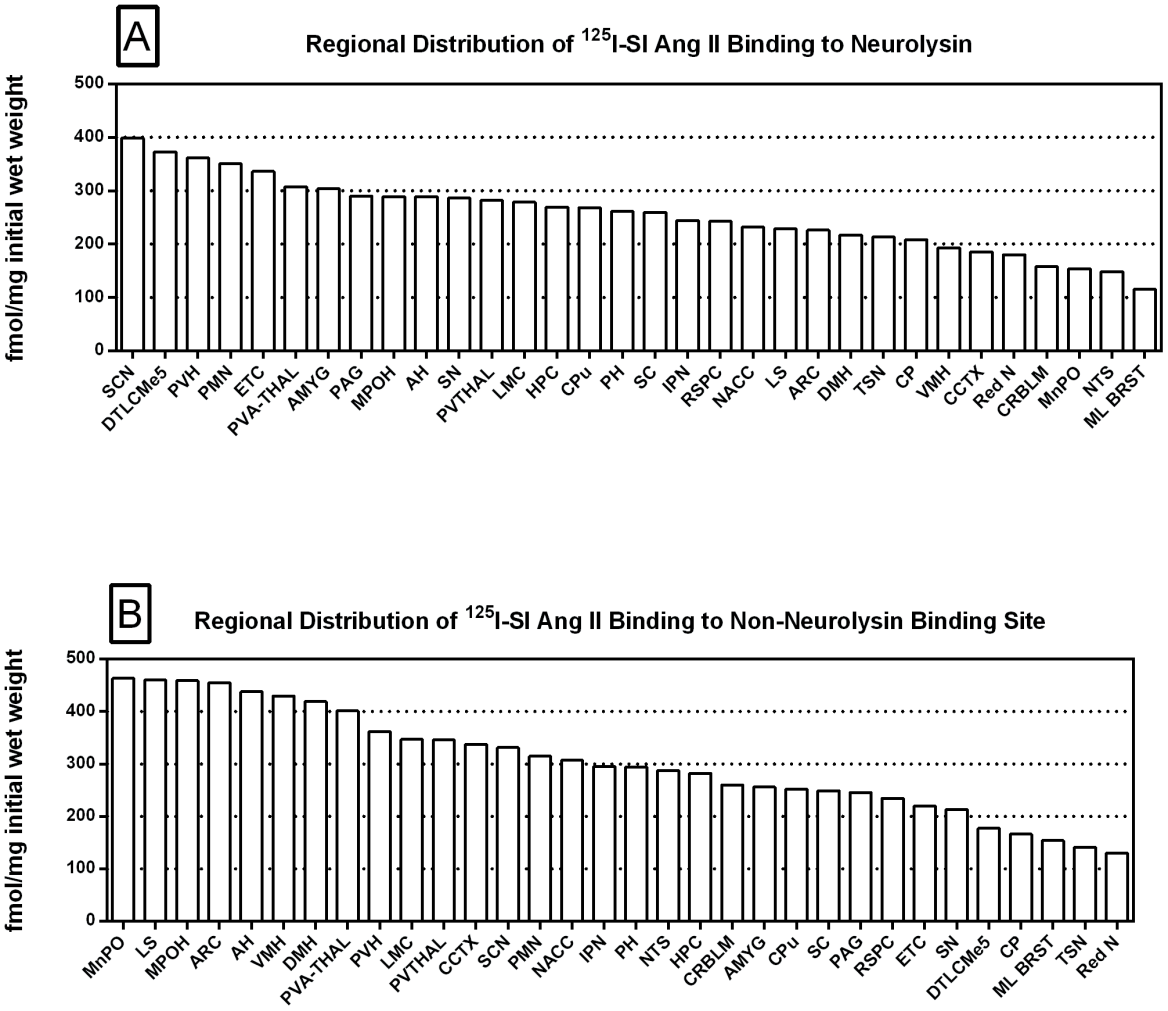
Regional distribution: neurolysin and non-AT_1_, non-AT_2_, non-neurolysin binding. Regional distribution of ^125^I-SI Ang II binding to neurolysin (Panel A) and non-AT_1_, non-AT_2,_ non-neurolysin (Panel B) in the mouse brain. Values represent the difference between non-AT_1_, non-AT_2_ Ang II binding in the WT and neurolysin KO mouse brains in 32 regions. AH, Anterior Hypothalamus; AMYG, Amygdala; ARC, Arcuate Nucleus; CCTX, Cingulate Cortex; CP, Choroid Plexus; CPu, Caudate Putamen; CRBLM, Cerebellum; DMH, Dorsomedial Hypothalamus; DTLCMe5, Dorsal Tegmentum, Locus Coeruleus, Mesencephalic Nucleus of the Trigeminal Nerve; ETC, Entorhinal Cortex; HPC, Hippocampus; IPN, Interpeduncular Nucleus; LMC, Limbic Cortex; LS, Lateral Septum; ML BRST, Mediolateral Brain Stem; MnPO, Median Preoptic Nucleus; MPOH, Medial Preoptic Nucleus; NACC, Nucleus Accumbens; NTS, Nucleus Tractus Solitarius; PAG, Periaqueductal Gray; PH, Posterior Hypothalamic Area; PMN, Premamillary Nucleus; PVA-THAL, Paraventricular Thalamic Nucleus, Anterior; PVH, Paraventricular Hypothalamic Nucleus; PVTHAL, Paraventricular Thalamic Nucleus; Red N, Red Nucleus; RSPC, Retrosplenial Cortex; SC, Superior Colliculus; SCN, Suprachiasmatic Nucleus; SN, Substantia Nigra; TSN, Triangular Septal Nucleus; VMH, Ventromedial Hypothalamic Nucleus.

Specific binding of ^125^I-SI Ang II to neurolysin was derived by subtracting specific binding in the neurolysin knockout brain regions from the specific binding in their counterpart wild-type brains (Figure 14, Panel A). Of the regions sampled, highest binding was found in the suprachiasmatic nucleus of the hypothalamus, and lowest binding was found in the mediolateral medulla. The difference in specific binding of ^125^I-SI Ang II to neurolysin between the highest and lowest regions surveyed was 3.5-fold.

Specific binding of ^125^I-SI Ang II to the non-AT_1_, non-AT_2_, non-neurolysin binding site is displayed in the rank order of highest to lowest binding (Figure 14, Panel B). There was no correlation in the density of non-neurolysin and neurolysin binding, R^2^ = 0.0036. Non-neurolysin binding was highest in the MnPO and other hypothalamic nuclei, the lateral septum and other frontal forebrain regions, and was lowest in the midbrain and brain stem regions. The variation in density from highest to lowest regions surveyed was 3.6-fold.

**Figure 15:**
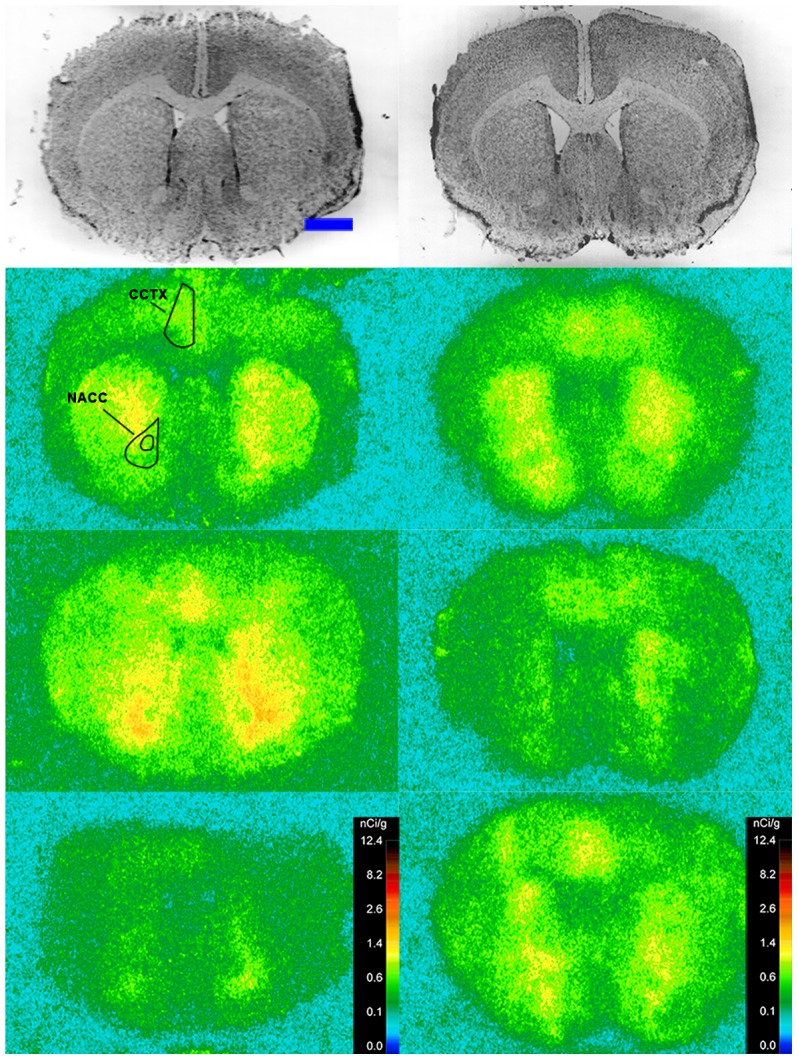
AT_1_ and AT_2_ receptor binding comparison. Comparison of ^125^I-SI Ang II binding to the AT_1_ and AT_2_ receptors of neurolysin KO (right panels) and WT (left panels) mouse strain brains in the presence of PD123319 or losartan, respectively. Approximate coordinates relative to Bregma: +0.98 mm (histology and autoradiograms) for KO, and Bregma +0.92 (histology), +0.86 (AT_1_), and +0.98 mm (AT_2_ and non-specific) for WT. Top row of panels are thionin-stained coronal sections adjacent to the sections used to generate the autoradiograms for “total” ^125^I-SI Ang II binding to the AT_1_ receptor (second row), AT_2_ receptor (third row), and “non-specific” ^125^I-SI Ang II binding (fourth row), represented in pseudocolor. The vertical calibration bar represents the relationship between binding density of ^125^I-SI Ang II and the color spectrum. The horizontal calibration bar in the upper left panel  = 2 mm. This pattern is repeated for Figures 16 and 17.

**Figure 16:**
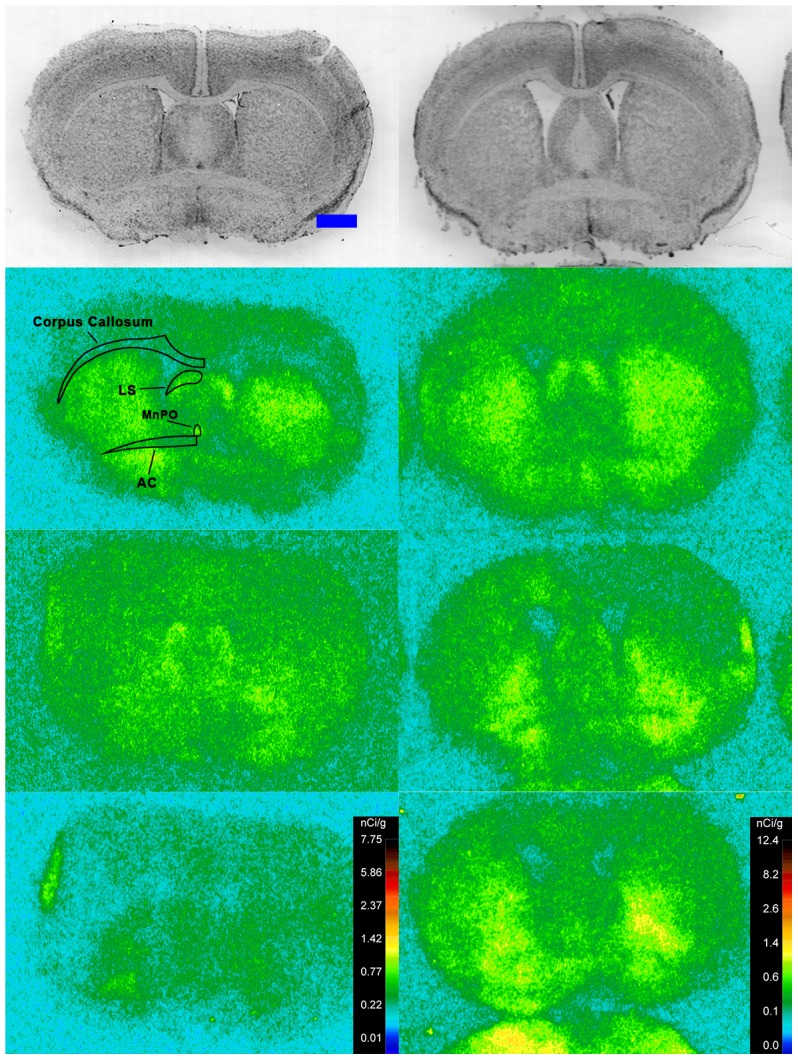
AT_1_ and AT_2_ receptor binding comparison. Comparison of ^125^I-SI Ang II binding to the AT_1_ and AT_2_ receptors of neurolysin KO and WT mouse strain brains in the presence of PD123319 or losartan, respectively. Bregma +0.14 (histology and AT_1_) and +0.02 mm (AT_2_ and non-specific) for KO, and +0.14 (histology, AT_1_, and non-specific) and +0.26 mm (AT_2_) for WT.

**Figure 17:**
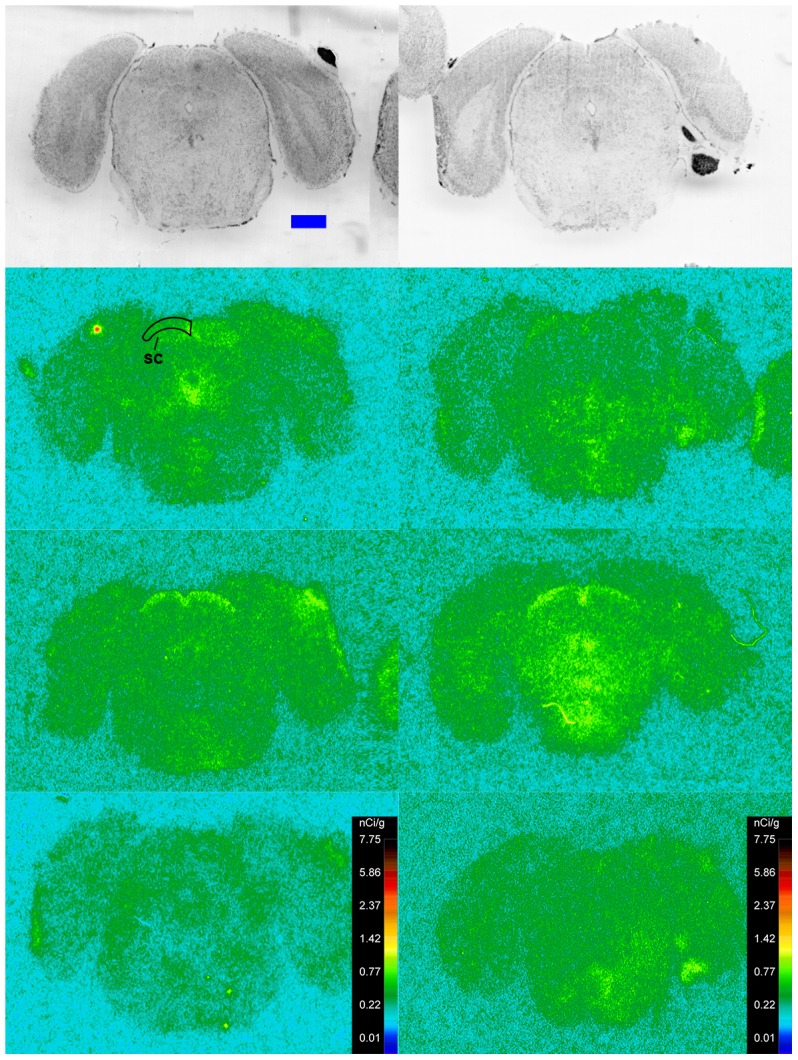
AT_1_ and AT_2_ receptor binding comparison. Comparison of ^125^I-SI Ang II binding to the AT_1_ and AT_2_ receptors of neurolysin KO and WT mouse strain brains in the presence of PD123319 or losartan, respectively. Bregma −4.48 (histology) and −4.36 mm (autoradiogram sections) for KO, and Bregma +4.36 mm for the WT histological and autoradiogram sections.

**Figure 18:**
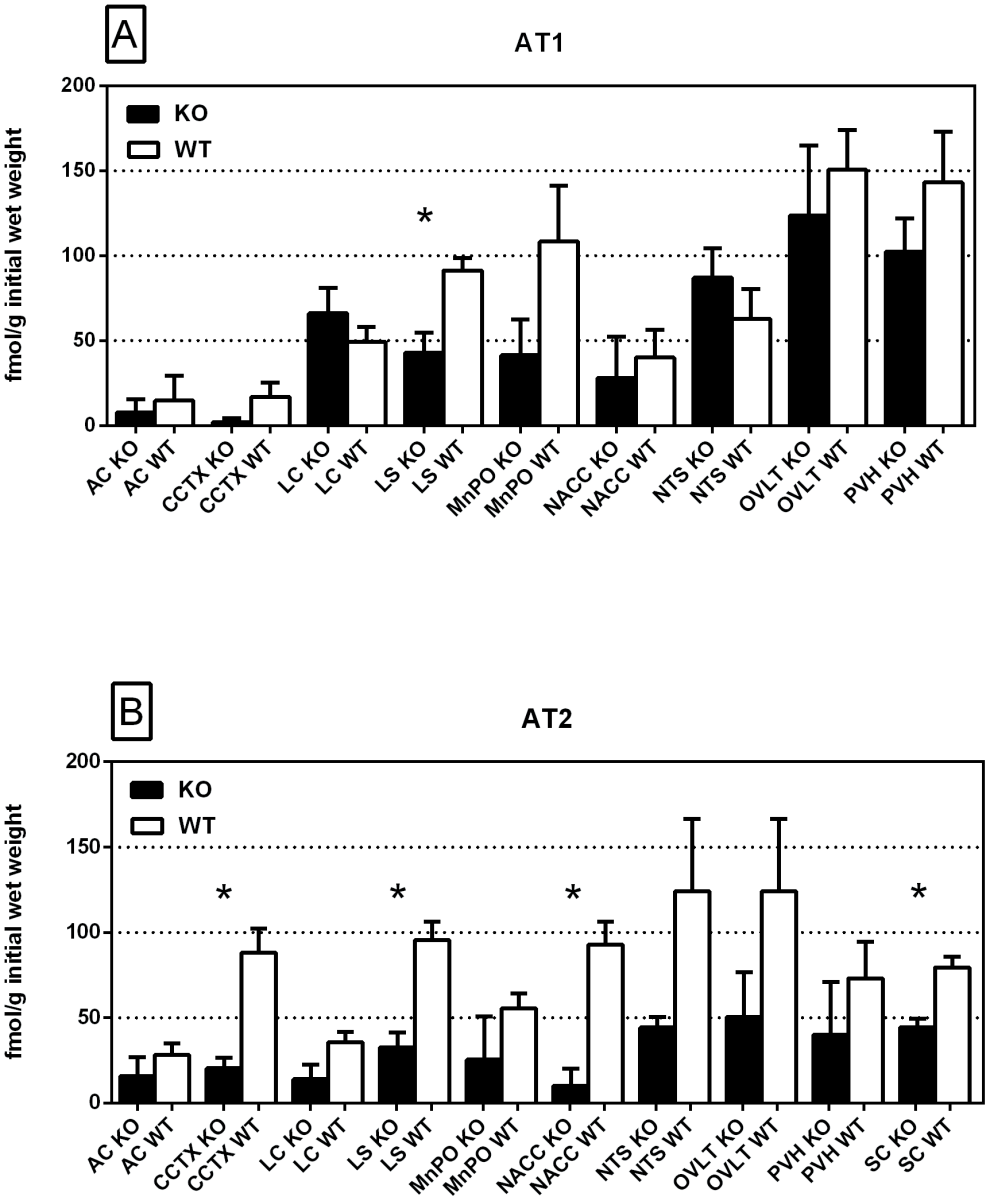
Regional distribution: AT_1_ and AT_2_ receptor binding. Regional distribution of ^125^I-SI Ang II binding to the AT_1_ and AT_2_ receptors in the neurolysin KO and WT mouse brains. Panel A describes binding to the AT_1_ receptor in 9 brain regions. Panel B describes binding to the AT_2_ receptor in 10 brain regions. * p<0.05. AC, Anterior Commissure; CCTX, Cingulate Cortex; LC, Locus Coeruleus; LS, Lateral Septum; MnPO, Median Preoptic Nucleus; NACC, Nucleus Accumbens; NTS, Nucleus Tractus Solitarius; OVLT, Organum Vasculosum of the Lamina Terminalis; PVH, Paraventricular Hypothalamic Nucleus; SC, Superior Colliculus.

Specific binding of ^125^I-SI Ang II to AT_1_ and AT_2_ receptors was observed in the presence of PD123319 or losartan, respectively (Figures 14–17). AT_1_ receptor binding was measured in 9 brain regions (Figure 18, Panel A). Two-way analysis of variance of AT_1_ receptor binding revealed a highly significant (p<0.0001) regional variation in binding density. There was a trend (p = 0.0597) towards reduced AT_1_ receptor binding (27%) in the brains of the neurolysin knockout mouse strain. Despite the appearance of increased AT_1_ receptor binding in the locus coeruleus and solitary tract nucleus area, there was no strain by region interaction (p = 0.477), indicating that the trend towards reduced AT_1_ receptor binding in the neurolysin knockout mouse strain did not vary significantly between brain regions.

AT_2_ receptor binding was measured in 10 brain regions (Figure 18, Panel B). Two-way analysis of variance of AT_2_ receptor binding revealed a highly significant (p<0.0001) regional variation in binding density. There was a highly significant (p<0.0001) reduction of 57% in AT_2_ receptor binding in the brains of the neurolysin knockout strain. There was no strain by region interaction (p = 0.536), indicating that the reduction in AT_2_ receptor binding in the neurolysin knockout mouse strain did not vary significantly between brain regions.

Quantitation of ^125^I-SI Ang II binding required a subjective evaluation of the adequate sample area of the region or nucleus of interest. Comparison of sampled area values for ^125^I-SI Ang II binding in the presence of PCMB between the wild-type and knockout strains showed an insignificant decrease of 0.4% in the neurolysin knockout strains across regions measured. A similar comparison of sampled area values for AT_1_ and AT_2_ binding between the wild-type and knockout strain showed a small increase of 2.3% and 2.2%, respectively, in the neurolysin knockout strains compared to the wild-type strain across regions measured.

**Figure 19:**
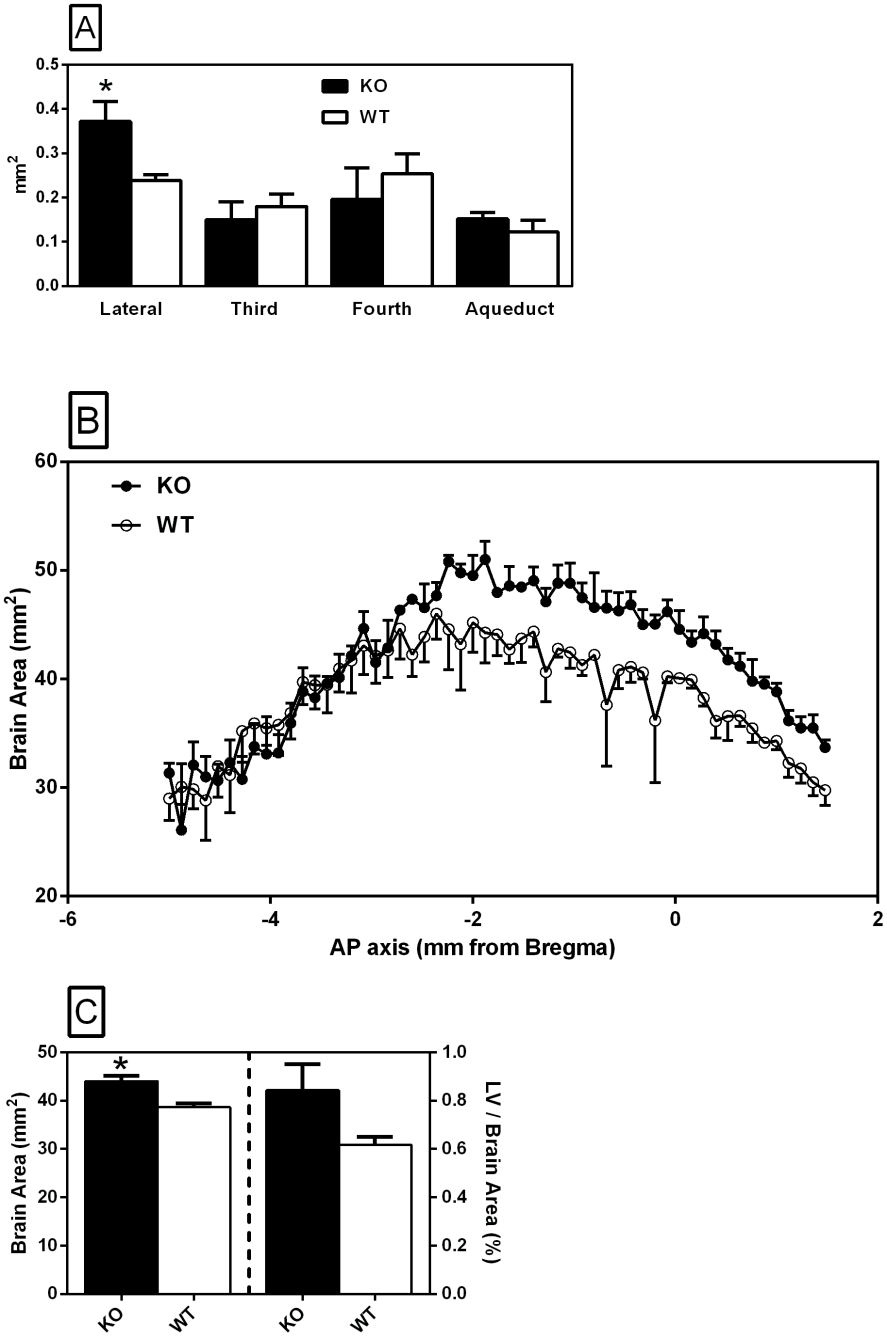
Ventricular and total surface area of brain sections. Comparison of ventricle size and total surface area of neurolysin KO and WT mouse strain brain coronal sections. Panel A describes the average surface area of lateral ventricles from the rostral limit of the hippocampus (∼0.9 mm caudal to Bregma) to the most rostral extent of the corpus callosum crossing (∼1.15 mm rostral to Bregma). Third ventricle surface area was measured from the rostral limit of the hippocampus (∼0.9 mm caudal to Bregma) to the rostral limit of the subfornical organ (∼0.2 mm caudal to Bregma). Fourth ventricle surface area was measured from ∼6.66 mm to ∼5.34 mm caudal to Bregma. Cerebral aqueduct surface area was measured from ∼4.84 mm to ∼4.24 caudal mm to Bregma. Panel B describes the average surface area of coronal brain sections at 120 micron intervals (5 mm caudal to Bregma to 1.48 mm rostral to Bregma). Panel C describes the average surface area corresponding to the sections used to measure the lateral ventricle area in Panel A (left Y-axis) and the ratio (%) of lateral ventricle size to total surface area of coronal brain sections (right Y-axis). * p<0.05.

Assessment of the lateral ventricle surface area between ∼1.5 mm caudal and ∼1.0 mm rostral to Bregma showed a significant (p<0.05) increase of 56% in the neurolysin knockout mouse strain (Figure 19, Panel A). There was no statistically significant difference in the surface area of the third and fourth ventricles, and the cerebral aqueduct, between knockout and wild-type strains (Figure 19, Panel A; p = 0.578, 0.530 and 0.387, respectively). Analysis of the total surface area of the coronal sections revealed a significant difference in cross-sectional area between the two strains (Figure 19, Panel B). Between 5.0 and 2.72 mm caudal to Bregma there was no apparent difference in surface area; however, from 2.72 mm caudal to 1.48 mm rostral to Bregma the total surface area of the knockout brain was on average 12% greater than that of the wild-type brain. Two-way analysis of variance (anterior-posterior (AP) axis and strain) revealed a significant AP axis by strain interaction (F_1,54_ = 1.69, p<0.005), as well as an expected AP axis main effect (F_4,54_ = 19.65, p<0.0001) with no significant strain effect (F_1,4_ = 3.66, p = 0.128). The average total surface area of the coronal sections from which the lateral ventricle size was determined (Figure 19, Panel C, left Y-axis) was significantly greater in the knockout strain (p<0.05). There was a nonsignificant (p = 0.115) tendency toward increased lateral ventricle size to total surface area ratio in the knockout brains (Figure 19, Panel C, right Y-axis).

## Discussion

The substantial decrease in ^125^I-SI Ang II binding in the presence of PCMB in the brains of the neurolysin knockout mouse strain confirms our previous observation that neurolysin is the non-AT_1_, non-AT_2_ Ang II binding site [40]. A definitive pattern of ^125^I-SI Ang II binding to neurolysin can be seen by subtracting out the ^125^I-SI Ang II binding in the neurolysin knockout mice from ^125^I-SI Ang II binding in the wild-type brains (Figure 14B). Neurolysin binding was widespread throughout the brain, showing only a 3.5-fold difference in density among sampled brain regions, in contrast to the discrete localization of AT_1_ and AT_2_ receptors in the mouse brain [53]–[56]. Indeed, neurolysin has a broad array of substrates [44], [45], thus its distribution beyond that of the angiotensin receptors is not unexpected. Noteworthy to its potential functional significance in the brain is its high expression in nuclei associated with circadian rhythms (suprachiasmatic nucleus), arousal (locus coeruleus), sympathetic nervous system activation (paraventricular hypothalamus), fear and anxiety (amygdala), Parkinson's disease (substantia nigra), Alzheimer's disease (hippocampus), and drug addiction (nucleus accumbens).

With respect to the functional significance of neurolysin to the brain RAS, the significant reduction in AT_2_ receptor binding suggests that neurolysin plays a role in maintaining AT_2_ receptor expression in the brain. There are two comprehensive studies of the regional density of mouse brain AT_1_ and AT_2_ receptors [53], [57]. While they show agreement with the regions that contain AT_1_ receptor binding, the relative densities in 7 overlapping regions were not significantly correlated. The distribution of AT_2_ receptor binding in this study varied from the comprehensive studies with respect to AT_2_ receptor binding in the hypothalamus [53], [57]. However a limited study of the hypothalamic AT_2_ receptor binding [54] as well as an immunohistochemical analysis [58] indicated the presence of AT_2_ receptors in the paraventricular nucleus of the hypothalamus in agreement with this study. The up-regulation of brain AT_2_ receptors is yet another indicator of a potential beneficial effect of neurolysin, since increased expression and/or stimulation of brain AT_2_ receptors is associated with neuronal protection [59], [60].

The lateral ventricular enlargement observed in the neurolysin knockout brains and the presence of neurolysin in the choroid plexus may indicate a role for neurolysin in the blood brain barrier and blood-cerebrospinal fluid permeability. This effect was limited to the lateral ventricles as no changes in the third and fourth ventricles or the cerebral aqueduct were observed between strains. This indicates that reduced flow of CSF through the cerebral aqueduct is not a cause of the lateral ventricle enlargement. Peptidases in the cerebral microvasculature and choroid plexus decrease the effects of circulating peptides on the cerebral microvasculature and help prevent blood-borne peptides from entering the brain via metabolic inactivation [61]. In the absence of neurolysin, its circulating peptide substrates may have more powerful actions on brain microvasculature circumventricular organs and the choroid plexus. There may even be an increased penetration of these peptides through the blood-brain or blood-CSF barrier allowing them to exert actions on periventricular brains structures, e.g., ependyma, leading to remodeling of the lateral ventricles. Ang II can damage the blood-brain barrier leading to hypertensive encephalopathy [62], [63]; this effect could be exacerbated by the loss of its metabolic inactivation by neurolysin. Future studies should be directed to determining if Ang II or another peptide substrate of neurolysin causes this lateral ventricle remodeling.

The pattern of neurolysin mRNA expression reported in the Allen Brain Atlas: http://mouse.brain-map.org/experiment/show/638735 using in situ hybridization [64] shows some similarities with the pattern of neurolysin binding reported in this study. Neurolysin mRNA expression is high in the pyramidal layer of the pyriform cortex, at the interface of layers 1 and 2 of the cerebral cortex and deeper layers of the frontal and entorhinal cortices; CA3 and dentate gyrus regions of the hippocampus; and the laterodorsal tegmental nucleus. High binding to neurolysin was detected in an area delineated as the dorsal tegmental, locus coeruleus, mesencephalic nucleus of the trigeminal nerve (DTLCMe5, Figure 11); the hippocampus (Figures 8 and 9) and the cerebral cortex (Figures 2–10), as summarized in Figure 14. However, some areas of high binding, e.g., the suprachiasmatic nucleus and the paraventricular nuclei of the thalamus and hypothalamus, show negligible neurolysin mRNA expression. These mismatches suggest that a significant proportion of membrane associated neurolysin is expressed on axon terminals distant from its site of synthesis in neuronal cell bodies.

Neurolysin has been shown to be present on the extracellular surface of cortical neurons and is therefore capable of metabolizing Ang I and Ang II in their extracellular environment [65], [66]. Moreover, formation of Ang (1–7) by neurolysin [45] diverts the conversion of Ang I into Ang II, directly counteracting the effects of the latter. While our studies have largely focused on membrane bound/associated neurolysin, neurolysin is also reported to be present in the mitochondria and cytosol [66], [67]. Indeed, the soluble angiotensin binding protein isolated from the liver is now known to be a cytoplasmically localized neurolysin [68]. Thus, neurolysin may play a role in the intracellular RAS [69] and other intracrine systems [70]. The importance of neurolysin relative to the other peptidases which metabolize Ang I and Ang II, shown in Figure 1, remains to be determined.

Neurolysin has the potential to play an important beneficial role in the RAS in four ways: 1) by forming a peptide, Ang (1–7), which counteracts the pathophysiological actions of Ang II, 2) by reducing formation of Ang II from Ang I by diverting Ang I away from ACE, 3) by metabolically inactivating Ang II in the extra- and intra-cellular milieu, and 4) by sustaining AT_2_ receptor levels in the brain. Future studies with neurolysin deficient mice and/or selective inhibitors of neurolysin to determine the levels of brain angiotensin peptides, blood pressure, thirst and salt appetite, and neurological phenotypes associated with the loss of neurolysin, should establish its functional significance.

Noteworthy in this study is the presence of a large amount of residual specific binding in the brains of the neurolysin knockout mice. This suggests the existence of a non-AT_1_, non-AT_2_, non-neurolysin Ang II binding site with a different pattern of expression in the mouse brain. A much smaller amount of ^125^I-SI Ang II binding (∼17% that of the wild-type strain) was observed in our previous study using membrane homogenates obtained from the brains of neurolysin knockout mice [40]. A possible explanation for this disparity may be the differences in tissue preparation. In preparing brain membrane homogenates the tissue is lysed, the membranes are precipitated centrifugally, and remaining components of the tissue (including the microsomal membrane fraction) are discarded with the supernatant. In contrast, the tissue sections used for receptor autoradiography contain all of the cellular membrane contents. It is possible that this non-AT_1_, non-AT_2_, non-neurolysin Ang II binding site remains in the supernatant of brain membrane homogenates and cannot be seen in membrane binding assays. Further studies to determine the identity of the non-AT_1_, non-AT_2_, non-neurolysin Ang II binding site and its affinity will be necessary to address this issue.

In conclusion, knockout of the neurolysin gene shows a significant effect on the RAS by decreasing ^125^I-SI Ang II binding to brain AT_2_ receptors. Additionally, neurolysin knockout mice display significantly enlarged lateral ventricles. The presence of substantial ^125^I-SI Ang II binding in neurolysin knockout brains in which classical Ang II receptors have been blocked suggests the presence of an additional non-AT_1_, non-AT_2_, non-neurolysin Ang II binding site of unknown function.
